# Isolation and freezing of human peripheral blood mononuclear cells from pregnant patients

**DOI:** 10.1016/j.xpro.2022.101204

**Published:** 2022-02-25

**Authors:** Athina Efthymiou, Nicoleta Mureanu, Rebecca Pemberton, Sarah Tai-MacArthur, Daniela Mastronicola, Cristiano Scottà, Giovanna Lombardi, Kypros H. Nicolaides, Panicos Shangaris

**Affiliations:** 1 Department of Women and Children's Health, School of Life Course & Population Sciences, Faculty of Life Sciences and Medicine King's College London, 10th Floor North Wing St Thomas' Hospital, London SE1 7EH, UK; 2 Peter Gorer Department of Immunobiology, School of Immunology & Microbial Sciences, Faculty of Life Sciences & Medicine, King's College London, London, UK; 3 Harris Birthright Research Centre for Fetal Medicine, King’s College London, London, United Kingdom

**Keywords:** Cell Biology, Cell isolation, Health Sciences, Clinical Protocol, Immunology, Stem Cells

## Abstract

To analyze immune cell populations accurately, a large number of Peripheral Blood Mononuclear Cells (PBMCs) must be obtained from blood samples. Traditional manual isolation and SepMate^TM^ isolation of PBMCs consistently yield blood-stained plasma layers and overall low numbers of CD4+ and CD8+ cells. Here, we describe an optimized protocol, using PBS with EDTA to increase PBMC yield from pregnant patients. This protocol enables analysis of CD4+, CD8+, and Regulatory T Cells and is potentially applicable to any immune cell population.

For complete details on the use and execution of this protocol, please refer to the SepMateTM website https://www.stemcell.com/products/brands/SepMateTM-pbmc-isolation.html.

## Highlights

• SepMate^TM^ Tubes offer fast and efficient PBMC isolation
• Using PBS with 1 mM EDTA increases the yield from pregnant patients’ blood samples
• Careful freezing ensures increased viability of cells
• Achieves increased viability by following a step-by-step thawing process

## Graphical abstract

**Figure:**
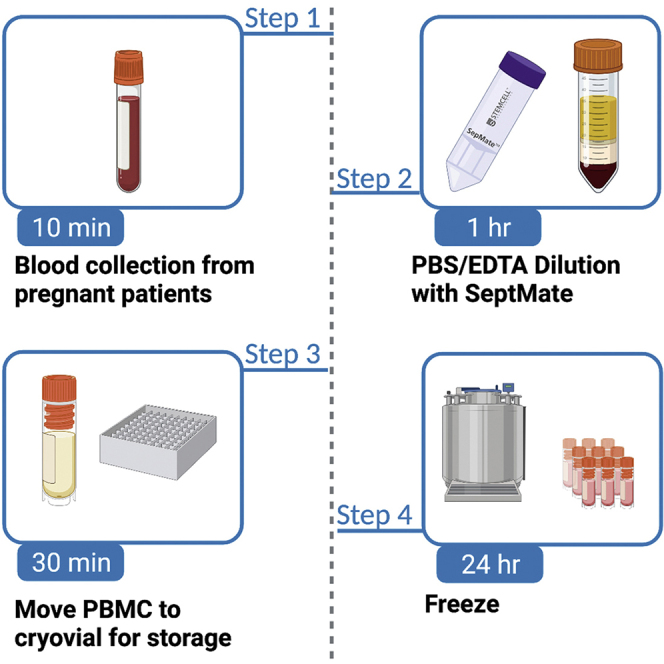

## Before you begin

The protocol below describes the specific steps for isolating and freezing PBMCs from pregnant patients. The challenge of the procoagulant state of pregnancy (Sanches et al., 2020) (including an increase in clotting factors, reduced anticoagulant and fibrinolytic activity) made density centrifugation difficult, as the plasma layer was stained with blood. We found that use of EDTA in PBS to the blood before centrifugation improved separation and yield. Though blood is collected in EDTA lined tubes as standard, without dilution with further EDTA, the plasma layer remained stained. This protocol was developed to analyze CD4+, CD8+ and Regulatory T Cell (Treg) populations, though this protocol may be useful for the analysis of any immune cell line in pregnancy.

### Acronyms

PBMCs: Peripheral mononuclear cells

PBS: Phosphate buffered saline

EDTA: Ethylenediaminetetraacetic acid

DMSO: Dimethyl sulfoxide

IL-6: Interleukin-5

INF-g: Interferon gamma

TNF-a: Tumor necrosis factor alpha

MNC: mononuclear cells

RPMI: Roswell Park Memorial Institute

### Recruitment of pregnant women (patients and blood collection)

**Timing: 10–15 min per sample**

1. Collect venous blood in BD Vacutainer® EDTA Tubes (10 mL)
  a. Measure the usable whole blood volume to the nearest 0.5 mL
2. Keep the blood tubes at room temperature (18°C–22°C) under gentle agitation using an orbital shaker (90–100 rpm) until processing, as this improves the viability of PBMCs obtained. The blood sample should be processed as soon as possible after drawing, ideally within 8–12 h.

**CRITICAL:** Patient samples should be collected in accordance with international review board rules, including appropriate recruitment and patient consent. This study was approved by the King’s College Hospital Research Ethics Committee, REC number is 02-03-033, dated 01/04/2003. All the experiments conform to the relevant regulatory standards. A total of 18 mL of blood in 2 × 9 ml EDTA tubes were taken from pregnant women at 35–36 weeks of gestation.

### General preparation

3. Keep Ficoll-Paque PLUS (GE Healthcare Pharmacia, Cat No: 17144003) at room temperature (18°C–22°C)
4. Bring the PBS/EDTA with 2% FBS to room temperature (18°C–22°C)

## Key resources table

**Table:** 

REAGENT or RESOURCE	SOURCE	IDENTIFIER
**Biological samples**		

18 mL of blood in EDTA tubes	Pregnant Women, 36 weeks, average age 32 years	n/a

**Chemicals, peptides, and recombinant proteins**		

Ficoll-Paque PLUS	GE Healthcare Pharmacia	Cat No: 17144003
Phosphate Buffer Saline (PBS) with 1 mM EDTA	Lonza	Cat No: BE02-017F
Fetal Bovine Serum, qualified, heat-inactivated,	Gibco™, Thermo Fisher Scientific	Cat No: 26140087
Cell Freezing Medium-DMSO	Sigma-Aldrich	Cat No: 6164
RPMI 1640 W/O L-GLUTAMINE	LONZA	Cat No: 733-1690
Corning CoolCell	CORNING	CAT No: CLS432001-1EA
Cryovial, 2 mL, external thread, natural cap, 14 × 14 predefined datamatrix code, linear barcode and human readable code	Greiner Bio-One Ltd.	126263-2DG
CountBright Absolute Counting Beads	Thermo Fisher Scientific	Cat No: C36950

**Software and algorithms**		

BD FACSDiva™ Software	BD Biosciences	BD FACSDiva™ Software
ELabNext Software	Elab Inventory	ElabNext Eppendorf Group

**Other**		

SepMate^TM^ 50 mL Tubes	Stem Cell Technologies	Cat No:154500
BD Vacutainer® EDTA Tubes (10 mL)	Fisher Scientific Ltd	Cat No: 10331254
Falcon 50 mL Conical Centrifuge Tubes	Thermo Fisher Scientific	Cat No:10788561
Falcon 15 mL Conical Centrifuge Tubes	Thermo Fisher Scientific	Cat No: 11507411
BD LSRFortessa™ System	BD Biosciences	BD LSRFortessa™ System

## Materials and equipment

**PBS/EDTA with 2% FBS:** PBS with 1 mM EDTA (Lonza Cat No: BE02-017F)) + 2% FBS (Thermo Fisher Scientific Cat No: 26140087). 10 mL of FBS in 500 mL PBS/EDTA. When FBS is added, keep it at 4°C, for a maximum of 5 days and use it at room temperature.

**Table:** 

Reagent	Final concentration	Amount
PBS/EDTA	PBS with 1 mM EDTA	490 mL
FBS	2%	10 mL

**Cell Freezing Medium**-DMSO: (Sigma Aldrich Cat No: 6164) to be stored in −20°C and at +4°C when open (keep on the ice during isolation)

**Corning CoolCell Freezing** 1°C/min cryo-freezing container (can hold 12 cryovials) (CAT No: CLS432001-1EA)

***Alternatives:*** The SepMate^TM^- 50 (Stemcell Technologies, Cat No: 154500) tubes were used for efficiency and time saving, though we found that their use did not significantly increase the number of PBMCs isolated. Manual separation, whereby layering the diluted blood and removal of the buffy coat is done by hand, may be performed. Proper technique is required to prevent contamination during layering and avoid disturbing the gradient when manually isolating the buffy coat layer after centrifugation.

## Step-by-step method details

### Isolation of PBMCs from peripheral blood

**Timing: 1 h**

This step describes how to isolate PBMCs from whole blood using SepMate^TM^ Tubes (Blood et al., 2016)

1. Invert Ficoll-Paque PLUS several times.
2. Place 17 mL Ficoll-Paque PLUS medium into the SepMate™-50 tube by carefully pipetting it through the central hole of the SepMate™-50 insert (Ficoll to be just above the insert).
3. Transfer the blood to a separate 50 mL Falcon tube in a sterile manner, i.e., working in the hood, using sterile pipetting technique.
4. Dilute the blood 1:1 in PBS-EDTA with 2% FBS, close the tube and mix by careful inversion
  a. (In our study): add 16.5 mL blood +16.5 mL PBS-EDTA 2% FBS to reach a total of 33)mL; if less than 16.5 mL of blood is available, you can mix it with PBS-EDTA in a 1:1 ratio
  b. SepMate™-50 is designed to process 4–17 mL of an initial blood sample.
5. Keeping the SepMate™ tube vertical, add the diluted sample by pipetting it down the side of the tube. The sample will mix with the density gradient medium above the insert. *Take care not to pipette the diluted sample directly through the central hole.*
6. Centrifuge at 1200*×g* for 20 min at room temperature, with the brake on 7 (out of 9).

***Note:*** Each tube contains four layers after the centrifugation, the yellow plasma layer on the top, the white MNC (mononuclear cells) layer, the Ficoll layer, and the red cell layer on the bottom.

7. Remove some of the plasma layer without removing the interface with the PBMCs, to allow better washing of the cells.
8. Pour off the top layer, which contains the enriched MNCs, into a new 50 mL Falcon tube. Do not hold the SepMate™ tube in the inverted position for more than 2 s as this may disrupt the filter within the SepMate^TM^ tube, which ensures the Ficoll and red cell layer stay at the bottom.
9. Register the cryovials using the barcode and record them as PBMCs.
10. Fill up the 50 mL falcon tube with RPMI 1640 medium (Thermo Fisher Scientific, Cat No: 733-1690) and carefully mix.
11. Centrifuge at 300*×g* at 20°C for 8 min and discard the supernatant.
12. Repeat steps 10 and 11 using 5–10 mL PBS-EDTA with 2% FBS.
13. Centrifuge for 8 min at 300*×g* at 20°C and remove as much of the PBS-EDTA with 2%FBS as possible by pouring quickly without disturbing the pellet.

### Freezing of PBMCs

**Timing: ∼ 24 h**

Cryopreservation of PBMCs is vital for ensuring the integrity of the cells remains high after thawing, supporting high cell viability and ensuring sample data is representative of the donors.

14. Count and resuspend the cells in 1 mL of freezing medium DMSO: (Sigma Aldrich Cat No: 6164)
15. Ensure the cryovials (Greiner Bio-One Ltd Cat No: 126263-2DG) are resting on ice or in an icebox
16. Aliquot cells into one barcoded cryovial, holding it on wet ice until freezing begins (within 5 min).
17. Freeze cells in the Corning CoolCell chamber (Cat No: CLS432001-1EA) below –70°C.
18. After at least 12 h, transfer the cells in cryo boxes and record their position.
19. Leave the cryo boxes in a −80°C freezer for 12–24 h (max four days), then transfer the cryovials into the liquid nitrogen tank.

## Expected outcomes

The described protocol provides a reproducible method for isolating PBMCs, including lymphocytes (T cells, B cells, and Natural Killer cells), monocytes, and dendritic cells in pregnant patients. After thawing, the number of PBMCs obtained were counted using CountBright Absolute Counting Beads (Thermo Fisher Scientific, C36950) as per the manufacturer's instructions, using the BD Fortessa cell analyzer (BD Biosciences) combined with the BD FACSDiva software (BD Biosciences). Our protocol yielded an average of 53 × 10^6^ PBMCs per healthy pregnant patient, though there will be biological variability in each individual.

**Figure 1:**
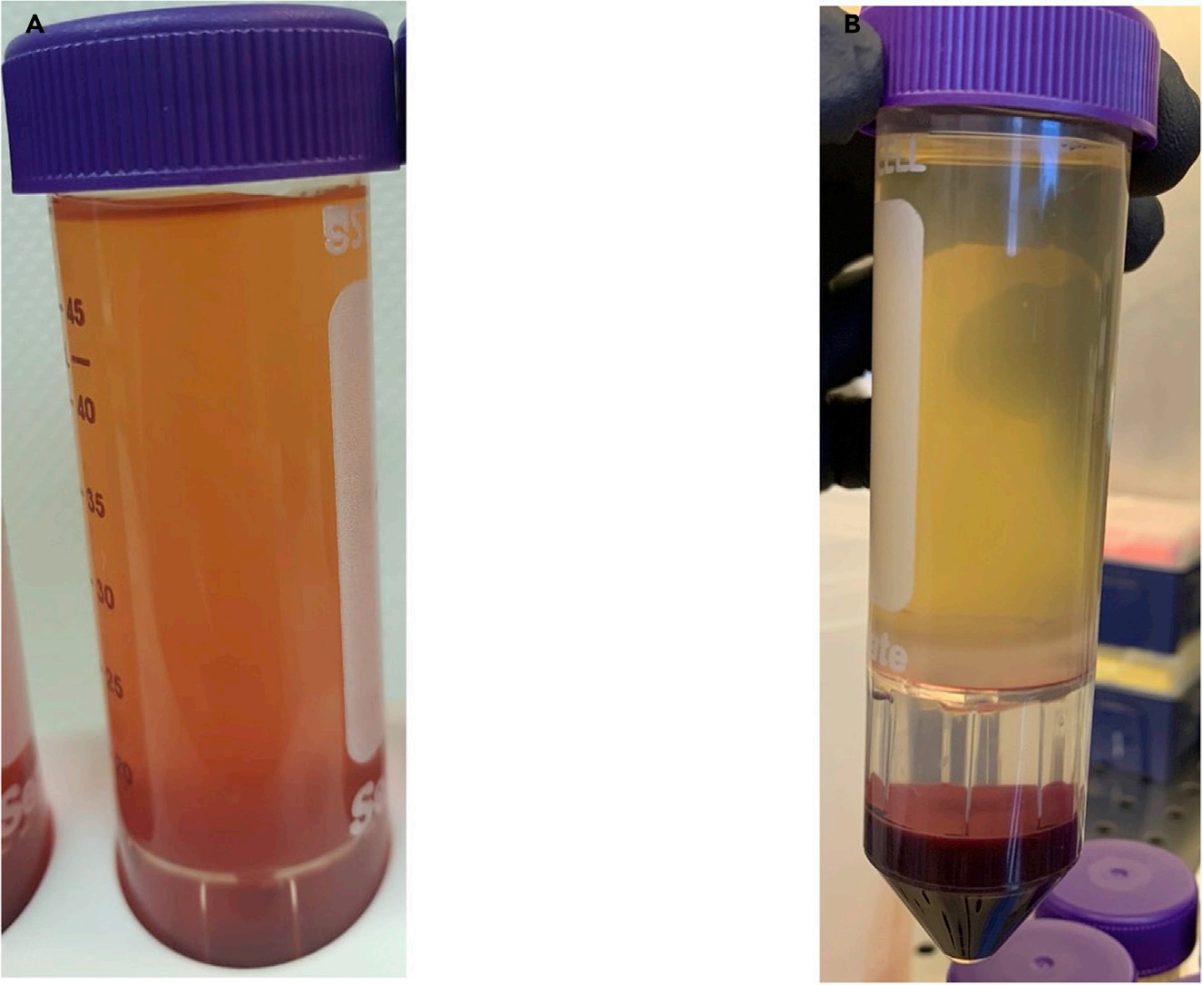
Comparison of cell separation using PBS and PBS EDTA Compared to diluting blood samples in PBS without EDTA (A), the addition of PBS/EDTA 1 mM removed the blood staining the plasma layer (B).

**Figure 2:**
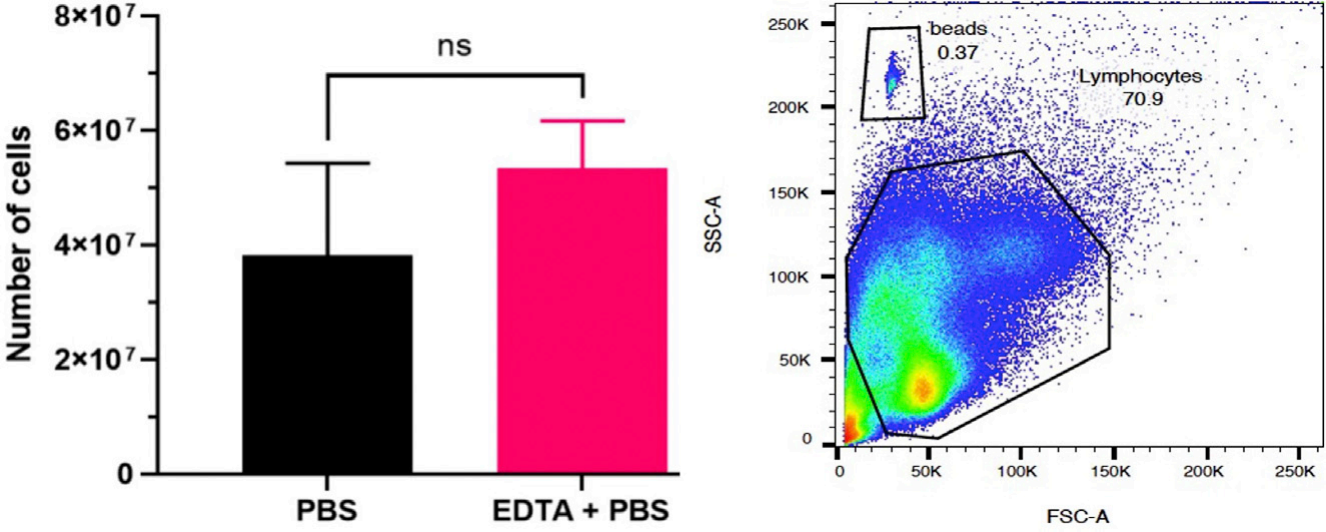
Flow Cytometric Analysis of T cells using PBS and PBS/EDTA 1 mM Left panel: PBMCs were isolated from whole blood samples taken from healthy pregnant volunteers. Blood was diluted in either sterile PBS (PBS) or PBS containing 1 mM EDTA (PBS + EDTA). Addition of EDTA to PBS increased the number of viable PBMCs obtained by an average of 15 × 10^6^ cells (Mean± Standard error of the mean, PBS: 38 × 10^6^ ± 16 × 10^6^, EDTA + PBS:53 × 10^6^ ± 8 × 10^6^ cells) Density centrifugation was completed using the SepMate^TM^ tubes. The number of PBMC obtained were counted using the CountBright Absolute beads (right panel) and compared between the two conditions.

**Figure 3:**
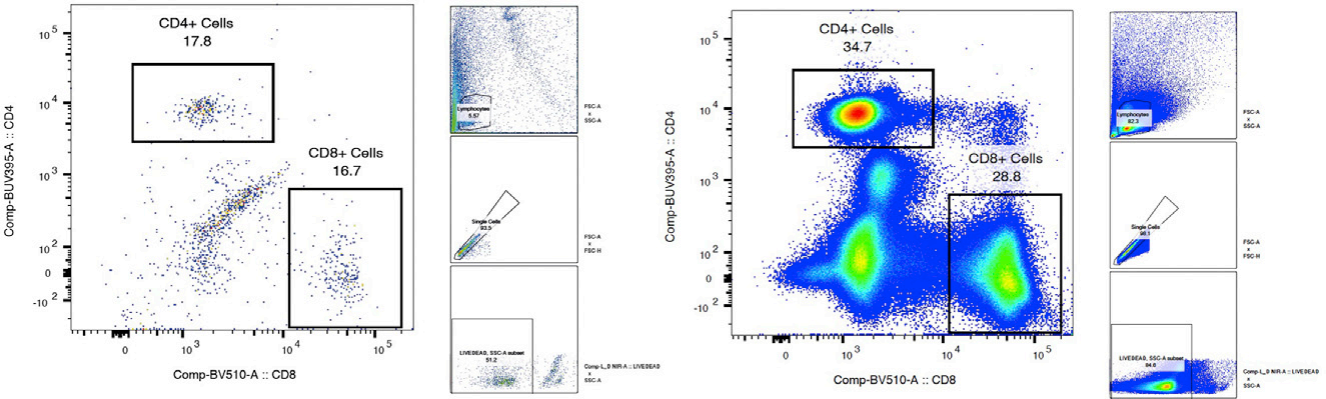
PBMCs were isolated from whole blood samples taken from healthy pregnant volunteers Blood was diluted in either sterile PBS (left panel) or PBS containing 1 mM EDTA (right panel). Density centrifugation was completed using the SepMate^TM^ tubes, and samples were frozen. Patient samples isolated using PBS or PBS containing EDTA were later thawed and analyzed using flow cytometry. Single live cells contained within samples were analyzed using the expression of CD4 and CD8 in the PBS sample to gate for CD4+ and CD8+ T cells and in an EDTA with PBS sample.

Protocols for isolation of PBMCs have been optimized in non-pregnant patients, and there is no current standard method for isolation of PBMCs in pregnant patients. The standard protocol of dilution of collected blood with PBS alone yielded low numbers of CD4+ and CD8+ T cells in our pregnant population. We noted that after density centrifugation, the plasma layer was blood-stained indicating potential contamination of the PBMCs. This can be seen in Figure 1, which demonstrates a comparison of diluted blood samples with PBS without EDTA (Figure 1A) and the addition of 1 mM of EDTA (Figure 1B). We considered the differences in haemostatic profile in pregnant women vs healthy controls – during pregnancy, the significant increase in clotting factors, reduced quantity of anticoagulants and reduced fibrinolytic activity creates a state of hypercoagulability (Sanches et al., 2020). This likely makes density centrifugation more complicated, and why the anticoagulant EDTA helped reduce blood contamination. The PBMCs obtained were counted using CountBright Absolute Counting beads and compared between the two conditions. In our case, the addition of EDTA to PBS increased the number of viable PBMCs obtained by an average of 15 × 10^6^ cells (PBS: 38 × 10^6^ ± 16 × 10^6^, EDTA + PBS:53 × 10^6^ ± 8 × 10^6^ cells) (Figure 2). The difference was not significant (*p*=0.2208), though upon analysis with flow cytometry, the addition of EDTA to PBS allowed a higher frequency of CD4+ T cells and CD8+ T cells to be isolated compared to PBS alone. (Figure 3)

## Limitations

We recognize this protocol has limitations. An area of consideration is the effect of cryopreservation on the viability and function of the PBMCs. In studies comparing frozen PBMCs vs fresh PBMCs, fresh PBMCs yield more viable cells (Weinberg et al., 2009). Ficoll isolation and cryopreservation affect the ratio of plasmacytoid to conventional dendritic cell subsets and their chemokine receptor expression. Some studies have also found frozen PBMCs to spontaneously secrete more IL-6, IFN-γ, TNF-α. (Mallone et al., 2011) While there is likely reduced viability and altered functioning, processing and freezing is sometimes necessary and allow for batch analysis of PBMCs at single laboratories, reducing confounding factors. Though there are differences in the absolute number of viable cells, results of assays performed using frozen PBMCs correlate well with fresh PBMCs, and differences when present are consistent across subjects. (Weinberg et al., 2009)

Another line of caution is that EDTA may not be the preferred anticoagulant for PBMC isolation for some applications. For example, it has been shown that natural killer cytotoxicity is dramatically decreased in EDTA, making it less suitable for NK cytotoxicity assays. Consideration of the impact of EDTA on the downstream analysis is required. (Betsou et al., 2019)

## Troubleshooting

### Problem 1

Incomplete separation of whole blood into layers (step 6).

### Potential solution

Ensure whole blood is fresh and uncoagulated (use of EDTA tubes, appropriate agitation, and storage at room temperature)

Reduce ejection speed of pipette and tilt SepMate^TM^ tube. This will help to layer the whole blood onto the Ficoll slowly.

### Problem 2

Decreased viability after freezing the cells (step 14)

### Potential solution

When you cryopreserve cells, make sure that you work quickly and efficiently

Always work on ice and avoid leaving the cells in the freezing medium at room temperature after re-suspension.

Always use cryopreserve cells at −1°C/min using the Corning CoolCell chamber (Cat No: CLS432001-1EA).

### Problem 3

Decreased viability after thawing the cells (when using own protocol)

### Potential solution

When thawing, make sure that you work fast and efficient

Submerge the cryovial with the PBMCs vial in a 37°C water bath for about 1 min.

Using the medium of your choice, add 1 mL of warmed (37°C) medium in the cryovial with the thawed PBMCs using a transfer pipette.

The thawed PBMCs can be poured in a 15 mL conical Falcon^TM^ tube (catalog number 11507411) with 5 mL of warmed (37°C) medium.

Rinse the cryovials once with 1 mL of medium.

Incubate for 5 min, in the 15 mL conical Falcon^TM^ tube (catalog number 11507411), in the 37°C water bath.

Centrifuge for 5 min at 300×*g* at room temperature.

Pour off the supernatant and proceed to further downstream applications.

## Resource availability

### Lead contact

Further information and requests for resources and reagents should be directed to and will be fulfilled by the lead contact, Dr Panicos Shangaris, Email: panicos.shangaris@kcl.ac.uk

### Materials availability

This study did not generate new unique reagents.

## Data Availability

The published article includes all datasets generated or analyzed during this study.
